# Isolated Third Nerve Palsy and Alternate Hemiparesis in Brain Stem Stroke: A Case Report

**DOI:** 10.22336/rjo.2026.17

**Published:** 2026

**Authors:** Rizaldy Taslim Pinzon, Marlyna Afifudin, Cornelius Ricky Nitema Ziliwu

**Affiliations:** 1 Neurology Department, Duta Wacana Christian University School of Medicine, Yogyakarta, Indonesia; 2 Ophthalmology Department, Duta Wacana Christian University School of Medicine, Yogyakarta, Indonesia

**Keywords:** Weber’s syndrome, oculomotor nerve palsy, brainstem stroke, CT = Computed Tomography, TCD = Transcranial Doppler, DSA = Digital Subtraction Angiography, LDL = Low Density Lipoprotein

## Abstract

A very rare sign of brain stem stroke is Weber’s Syndrome. Ipsilateral third nerve palsy and contralateral hemiparesis are the hallmarks of Weber’s Syndrome. Midbrain infarction is the most frequent cause of Weber’s Syndrome. The perforating branches of the basilar bifurcation or occlusion of the posterior cerebral artery commonly cause the infarction. We report the case of a 58-year-old female diagnosed with Weber’s syndrome. The risk factors were hypertension and dyslipidemia. This patient presented with acute right-side motor weakness and left medial gaze palsy (third nerve palsy). A brain Computed Tomography (CT) scan revealed a lesion on the right side of the mesencephalon, which was consistent with an acute brainstem infarction. Subsequent Transcranial Doppler (TCD) and Digital Subtraction Angiography (DSA) revealed severe stenosis of the posterior circulation. We diagnosed and treated this patient as having Weber’s syndrome based on the symptoms, signs, and imaging findings.

## Introduction

Brainstem stroke syndrome is a relatively uncommon syndrome compared with supratentorial region stroke. The epidemiological data showed that it is very unusual for a midbrain infarction to cause Weber’s Syndrome [1]. In many previously reported cases, clinical findings may be associated with infarction in other areas of the vertebrobasilar system. Previous epidemiological studies have shown that isolated midbrain infarction accounts for less than 1% of cases of posterior circulation strokes [1,2].

This manifestation of stroke syndrome is very uncommon. Only a few case reports have mentioned the classic figure of Weber’s syndrome [3-5]. We report the case of a 58-year-old female with acute right-side motor weakness and left-side medial gaze palsy (Third Nerve Palsy).

## Case report

**Fig. 1:**
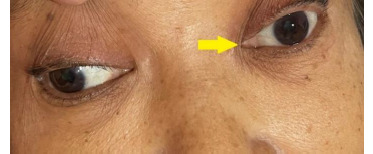
Left side oculomotor palsy with contralateral limb weakness

A 58-year-old female with a long history of uncontrolled hypertension and high cholesterol came to our emergency department, presenting with an 18-hour history of sudden right-side motor weakness and left eye deviation (oculomotor cranial nerve palsy) (**Fig. 1**). A Glasgow coma rating of 15/15 (eye-opening: 4, verbal response: 5, motor response: 6) indicated that the patient was completely cognizant and awake during the evaluation. The patient’s muscle strength was 4/5 on the right side, indicating right-sided weakness. Pupils were spared despite oculomotor palsy because they were equal in both eyes and reactive to light. There were no associated signs and symptoms of dysphagia, dysarthria, aphasia, bulbar paresis, or any other cranial nerve palsies. In the affected limb, the Babinski reflex was positive. The results of the other neurologic tests were normal.

**Fig. 2:**
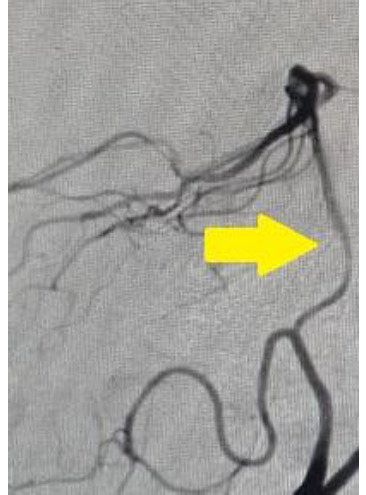
Digital subtraction angiography

Aside from uncontrolled hypertension and high lipid profile, the patient had no other significant medical or surgical history. Patient had no history of neurological disease (stroke, coagulopathy, limb weakness, ischemic heart disease. No significant history of prior medication or anticoagulation was found. The vital signs were normal, except for high blood pressure (170/100 mmHg). The patient’s score on the National Institute of Health Stroke Scale (NIHSS) was 6. The laboratory test results, which included a complete blood count and a broad biochemistry panel, showed very high LDL cholesterol (194 mg/dL) and a random blood glucose (186 mg/dL). The results of every other test, including echocardiogram, electrocardiogram, and cardiology consultations, were normal. The ophthalmology consultation revealed the diagnosis of third nerve palsy. The brain CT scan showed an acute infarction in the midbrain. The subsequent transcranial Doppler and digital subtraction angiography showed severe stenosis in the left vertebral artery, with the possibility of hypoplasia in the left vertebral artery (**Fig. 2**). Based on the clinical and radiological findings, Weber’s syndrome was diagnosed. The patient was treated with a loading dose of clopidogrel (300 mg) and continued dual antiplatelet therapy (low-dose aspirin 80 mg and clopidogrel 75 mg), folic acid, and a high-intensity statin (atorvastatin 40 mg daily). After physical therapy and 7 days of hospitalization, this patient was discharged with mild disability (Modified Rankin Scale: 2).

## Discussion

**Fig. 3:**
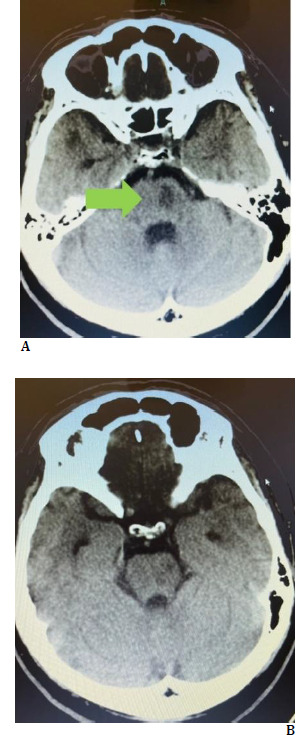
**A, B** Infarction in the midbrain

**Fig. 4:**
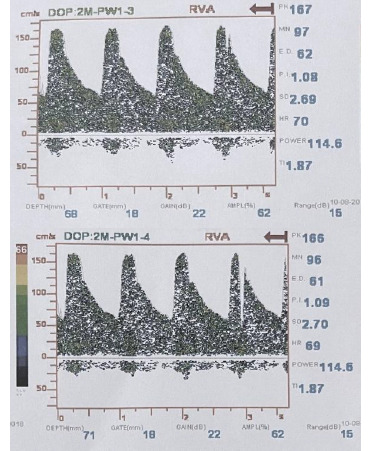
Severe stenosis in the left vertebral artery based on TCD (Mean Flow Velocity: 97 m/s)

We reported a very rare case of stroke as Weber’s syndrome. This case was caused by an ischemic lesion in the ventromedial midbrain (**Fig. 3, 4**) with very severe stenosis in the left vertebral artery. This syndrome is caused by disruption of the paramedian mesencephalic branches (basilar), the peduncular perforating branches (posterior cerebral artery), the choroidal arteries, and the superior cerebellar artery that supply the midbrain, which occurs with an isolated brain supply due to occlusion. In addition, other less common causes of this syndrome are hemorrhage, aneurysms, cavernomas, tumors, and demyelinating diseases [3,4].

It is very rare for a solitary midbrain infarction to cause Weber syndrome. In most cases, arterial occlusion in the vertebrobasilar system occurs at the same time as the infarctions in other areas. Patients with Weber’s syndrome present with several signs and symptoms, including ptosis and diplopia associated with contralateral partial or complete paralysis of the upper and lower limbs. In this syndrome, the hallmark neurological finding is crossed sensory disturbance or motor weakness, as well as oculomotor nerve palsy [2,3]. In this case, the patient had right-side motor weakness, medial gaze palsy (third nerve palsy that spares the pupil), and an acute onset. The patient’s condition improved gradually with medical treatment and physical therapy.

The third nerve nuclei are in the midbrain and extend approximately 10 mm from rostral to caudal. Most of the fiber in the pupils is supplied by the Edinger Westphal nucleus in the upper midbrain, while most of the fiber in the extra-ocular eye muscles is supplied by the motor nucleus in the lower midbrain [3,4]. Pathological lesions in the lower midbrain impair extra-ocular muscles but not pupils, whereas lesions in the upper midbrain cause pupillary dilatation [4,5]. This mechanism explains why this patient had pupillary sparing in Weber’s syndrome despite having a third nerve palsy. Most patients with Weber’s syndrome are neurologically stable [6,7].

The most important factors affecting the neurological outcome of these patients are early detection and care, adequate therapy to prevent complications, and precautions to prevent the recurrence of similar strokes. These are all essential elements in managing Weber’s syndrome [5,6,8]. Despite having a midbrain infarction, the patient in this case was stable and had moderate to mild disability at hospital discharge. We treated this patient appropriately; the condition gradually improved with medical treatment and physical therapy.

## Conclusion

We reported a very rare case of alternans infarction stroke presenting with right-side hemiparesis and left oculomotor palsy, sparing the pupil. A midbrain infarct was the cause of this syndrome, which is related to Weber’s syndrome.
